# The Influence of HEV-Filtering Contact Lenses on Behavioral Indices of Glare

**DOI:** 10.1097/ICL.0000000000000944

**Published:** 2022-10-06

**Authors:** Lisa M. Renzi-Hammond, John Buch, Jie Xu, Billy R. Hammond

**Affiliations:** Department of Health Promotion and Behavior (L.M.R.), Institute of Gerontology, University of Georgia, Athens, GA; Research & Development (J.B., J.X.), Johnson & Johnson Vision Care Inc, Jacksonville, FL; and Behavioral and Brain Sciences Program (B.R.H.), Vision Sciences Laboratory, University of Georgia, Athens, GA.

**Keywords:** Positive dysphotopsia, HEV-filter, Contact lenses

## Abstract

We assessed the effects of a HEV-filtering contact lens on positive dysphotopsia (halos and starbursts) and a behavioral index of scatter measured using two-point light thresholds. These effects were assessed by direct comparison to a clear (i.e., non-HEV filtering) contact lens tested in the fellow eye.

**Methods:**

Sixty-one subjects were randomized and fit with study lenses and 58 subjects completed the study. A double-masked contralateral design was used. Subjects were randomized to test lens-OD, control lens-OS, or vice versa. Participants were exposed to a point source of broadband simulated sunlight (a 403-nm condition was also tested) that created the appearance of halos/starbursts. The degree of dysphotopsia was measured as the diameter of broadband and violet-induced halos, and broadband light-induced starbursts. Two-point thresholds were assessed as the minimum resolvable distance between two pinpoints of light.

**Results:**

The HEV-filtering lens was statistically superior (*P*<0.0001) to the clear lens in all the conditions tested. The HEV-filtering lens significantly reduced halo diameter by 30%, starburst diameter by 23%, and resolvable distance in the two-point condition by 18% (white) and 30% (violet).

**Conclusions:**

HEV-filtering contact lenses can reduce some deleterious effects of bright broadband light by decreasing light scatter, halos, and starbursts.

The trichromatic vision of humans is based on cone types with distinct sensitivity to staggered regions of the visible spectrum. These cone types, short-wave (S), mid-wave (M), and long-wave sensitive (L), have many distinct properties. One class, however, appears to be categorically different.^1,2^ M and L cones appear to have a more recent evolutionary lineage, whereas S-cones are older and distinct.^3^ S-cones are numerically sparse (approximately 7%),^4^ absent in the central foveola, sluggish, and likely contribute only minimally to spatial vision.^5,6^ The preferential loss of S-cones is often used as an early indicator of retinal disease (e.g., Cho et al.^7^). Unlike the light most strongly stimulating M-and-L cones (500–720 nm), short-wave light (380–500 nm) is strongly filtered by the anterior lens and macular pigments. Widening our color vision to include the perception of short-wave light was a clear advantage, but blue objects are relatively rare in nature (e.g., only ∼10% of 300,000 flowering plants are HEV-filtering blue^8^), so this system could likely be minimized compared with the other chromatic mechanisms. S-cones are often not found in aquatic mammals and many nocturnal terrestrial species.^9^

Why is our underlying visual response to the lower third of the visible spectrum (so-called high-energy visible [HEV]: 380 to 500 nm^10^) reduced when compared with the longer-wave portions (>500 nm)? One argument might be that HEV light is actinic^10,11^ and the system evolved mechanisms to protect itself against such stress. Unlike ultraviolet that is absorbed by the cornea and lens, HEV light penetrates to the retina and is sufficiently energetic that HEV light can initiate photochemical damage (unlike mid-and-long wave light).^11^ The retinal pigment epithelium contains a number of photosensitizers (e.g., A2E^12^) that peak in the HEV region (even photopigment can serve as a photosensitizer, so having less photopigment absorbance of energetic light makes sense^13^).

Another possibility might be more directly functional. HEV light in the atmosphere is prone to scatter (consistent with Rayleigh's equation) and likely interferes with many aspects of visual function. For example, blue haze likely limits the ability to see objects in the distance.^14^ Visual functions, such as glare discomfort, have been shown to have action spectra that, even at equal energy, are exacerbated for HEV light.^15^

If HEV light mostly serves to enhance color vision (and nonvisual functions such as circadian rhythms) but is pernicious in other ways, then it would make sense that the visual system would evolve to minimize its deleterious optical effects and actively filter (e.g., Widomska et al.^16^). McLellan et al.^17^ originally showed that natural monochromatic aberrations reduced what otherwise would be large blurring effects (such as violet penumbras) because of chromatic aberrations that are exaggerated for HEV light. Short-wave absorbing intraocular filters are common in nature.^18^ Macular pigment, for example, can absorb more than 90% of HEV light with no effects on sensitivity or color vision.^19,20^ If the system is optimized to use HEV light that is carefully regulated, it is likely meaningful that modern exposure to HEV light is less predictable (i.e., not based simply on diurnal cycles because of artificial lighting) and minimizing has likely decreased (e.g., average MP levels have likely decreased because of decreased intake of green leafy vegetables^21^). Many devices and artificial lights now emit significant amounts of HEV light.^22^ Dietary intake of lutein and zeaxanthin is relatively poor in the modern diet^23^ and are likely less than historical norms. Many individuals work inside and likely have less yellowed (SW-absorbing) crystalline lenses than may once have been typical when jobs were mostly outdoors with excessive sun exposure.^24^

In a previous study,^25^ we showed that photochromic contact lenses reduce halo and starburst diameters and the distance necessary to resolve as separable two pin points of light. These photochromics have a relatively flat (neutral density) profile of filtering. In this study, we assessed whether only filtering the HEV portion of broad band light would produce similar effects. HEV-absorbing contact lenses only filter a relatively small proportion of the overall visible spectrum. Hence, a similar effect would assume that the HEV portion is driving a disproportionately high proportion of the deleterious visual effects to a broad-band light stimulus (emulating sunlight).

## METHODS

### Participants

**FIG. 1.**
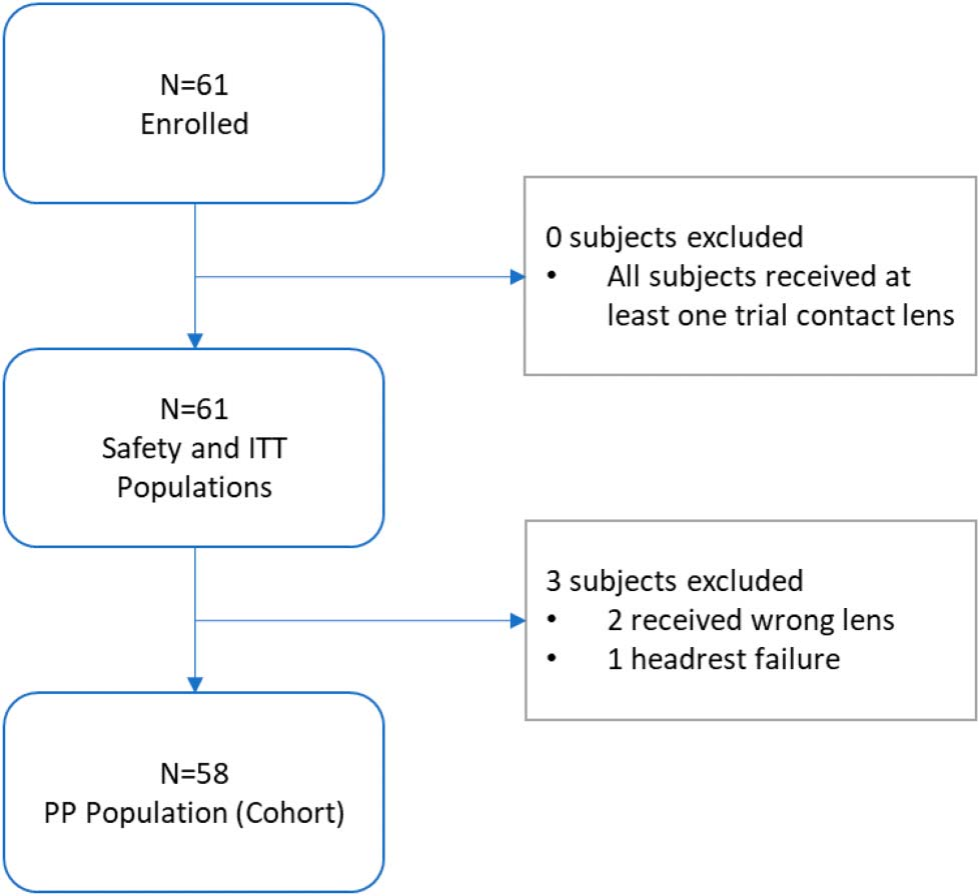
Subject accountability.

Participants were habitual wearers of spherical silicone hydrogel soft contact lenses with best-corrected visual acuity of 20/25 or better in each eye. A basic clinical examination was done to exclude any overt ocular pathologic condition (no subjects needed to be excluded). A total of 61 subjects (M=39.6±12.21 years; 77% female; 31.1% Black/African American; 68.9% White/Caucasian) were randomized and included in the intent-to-treat (ITT) population. Participants were fitted with study lenses and tested at a single clinical site (Georgia Center for Sight, Greensboro, GA). Among the 61 ITT subjects, 58 subjects (M=39.2±12.34 years) completed the study without a major protocol deviation and were included in the per-protocol population (Fig. 1 for subject accountability).

### Ethics

The study protocol and study-related materials were approved by Sterling IRB (Atlanta, GA). Verbal and written informed consents were obtained before testing. The tenets of the Declaration of Helsinki were adhered to at all times while conducting the study.

### Study Design

A prospective, randomized, double-masked, contralateral design was used. There are a couple benefits to this choice of design. First, subject factors that can influence visual performance, such as iris color and absorption of test lights by macular pigment, are better controlled using this design. Second, in psychophysical testing, which is the gold standard for many of the visual functions being tested, participants make judgments about some event threshold, such as when an image disappears or how large or bothersome or intense a visual event appears. Participants may have different criteria for threshold events that are internally consistent within subjects but can vary between subjects. A contralateral design allows the investigational lens to be compared within subjects, with consistent criteria for threshold events.

### Masking and Randomization

This was a double-masked study. Subjects were unaware of which lens (HEV-light filtering or clear control) was assigned to which eye (OD or OS; one lens randomized to each eye). The investigator conducting the psychophysical testing was also not informed about which eye was fitted with which lens (lens type on eye was not visually discernible). Instead, lens type was identified by code only, and the link between the code and the lens type was not revealed to the investigator conducting the psychophysical testing until after the database had been locked. The clinician fitting the lenses was not masked.

### Study Lenses

**FIG. 2.**
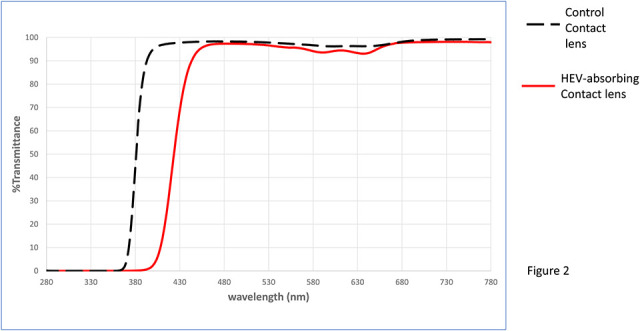
The transmission characteristics of the clear control contact lens and the HEV-absorbing test contact lens.

The test lens was a senofilcon-A prototype that filters approximately 60% of short-wave visible light between 380 and 450 nm. The control lens was a commercially available senofilcon-A lens that did not contain the HEV filter (a.k.a. “clear”), although senofilcon-A lens still contains a visibility tint. Lens powers were available from−1.00 through−6.00 D, and powers were adjusted until a plano spherical overrefraction was achieved OD and OS. The transmission characteristics of the test and control lenses are shown in Figure 2.

### Apparatus and Procedure

A schematic of the apparatus that was used is shown in Hammond et al.^25^ A 1000 W Xenon arc lamp was used as the light source (emulating white sunlight; Chromaticity Coordinates, x=0.33, y=0.329). An achromatic lens was used to partially collimate the light so that it provided homogeneous back illumination of an opaque light baffle. This baffle contained a small (4 mm) aperture, which created the primary stimulus. Halo and starburst diameters were measured using broadband light. In addition to measuring two-point thresholds using broadband light, a 403-nm interference filter was placed in the collimated portion of the light before passing through the light shield. This wavelength is strongly absorbed by the HEV-filtering lens and was used as a comparison with the broadband illumination condition.

Photometric calibrations were performed using an ILT 950 spectral radiometer (International Light Technologies, Peabody, MA). Radiometric calibrations were performed using a Graseby Optronics United Detection Technology instrument (Orlando, FL). The same United Detection Technology instrument was used periodically to ensure that the total light output of the optical system remained constant and consistent throughout the study. For all psychophysical tests described below, an eye patch was used to cover the nontest eye. Participants' heads were stabilized using an adjustable chin and forehead rest assembly.

### Measuring Halo and Starburst Diameters

**FIG. 3.**
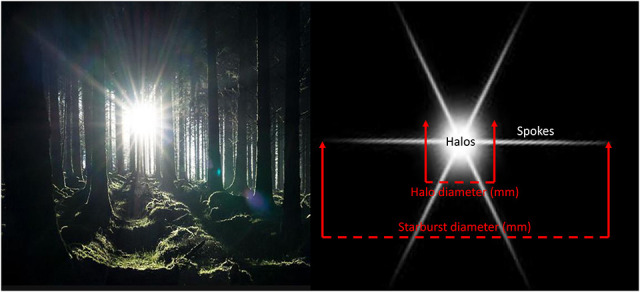
A naturalistic example of spokes and halos.

Before testing, the task was explained using pictorial examples of how natural halos and starbursts look to a typical observer (e.g., car headlights, streetlights, against a blue sky or created through stadium lights), along with images that showed the measurement procedure. An example of an image is shown in Figure 3. An aperture (4 mm at the light shield; approximately 14 min visual angle) was used to create a bright point source of light 38 inches from the plane of the eye (although it appears bright, the actual luminance of this small point of light is relatively low, e.g., 140 cd/m^2^ for the broadband measured at the aperture). Between the point source and subject, a centering precision caliper (approximately 23 inches from the plane of the eye) was used to measure lateral spread of halos (diffusion around the source) and visual spokes of the resulting starburst. For the caliper guides to be clearly seen by the subjects, the guides were covered with reflective material.

The ascending and descending method of limits was used to determine the diameters of the halo and starburst. A single trained experimenter moved the caliper guides until subjects indicated that the guides just surrounded the halo and the starburst (three trials were collected for each condition). The testing eye order (OD start or OS start) was randomized.

### Measuring Two-Point Thresholds

**FIG. 4.**
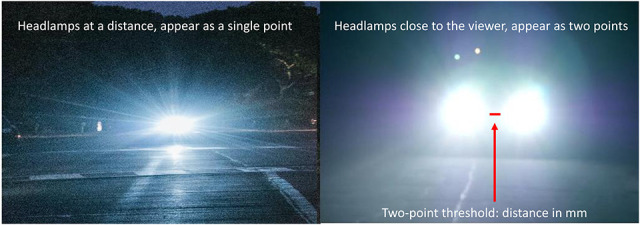
An ecological example of the two-point light separation task. The two headlights in the distant vehicle are fused and separate as the vehicle becomes closer to the observer.

The 4-mm aperture used for the halo/starburst measurement seen as a single point of light could be separated into two small (2 mm) apertures. These two small points of light were slowly moved apart until the stimulus was perceived as two separate points. A digital micrometer on the back of the light shield quantified the amount of separation needed. A pictorial example of the two-point threshold discrimination is shown in Figure 4.

Light from the point source (i.e., light emerging from the two 2-mm apertures in the light shield) was focused by a long (220 mm) focal length lens (12 inches from the plane of the eye). This lens was used to fuse the two test points so that the initial image was clearly seen as a single point (this could also have been accomplished with greater distance; however, given the need to measure in the eye clinic, space in the test room was limited). When the points were maximally close together, the stimulus appeared as a single, bright point of light (as the stimulus did when measuring halos and starbursts). The two apertures were slowly moved apart from this “zero point,” and subjects indicated when the spread from each light point did not overlap (e.g., when the subjects first perceived a small black space between the two points). A total of six trials were completed per test eye: three trials were completed using the broadband stimulus, and three trials were completed using the short-wave test condition. Again, test eye order was randomized.

### Statistical Analysis

Statistical analyses were performed using SAS version 9.4. All randomized subjects were analyzed according to the treatment to which the subjects were randomized after the ITT principle for testing statistical superiority.^26^ Starburst, halo, and the two-point threshold with and without 403-nm filter were analyzed separately using a generalized linear mixed model with a lognormal distribution. Subject demographics and baseline characteristics adjusted in the generalized linear mixed model analyses included age, dominant eye, gender, race, iris category (dark iris and light iris), and light sensitivity score (on a 0–100 scale). The correlation among within-subject repeated measures taken from different eyes was appropriately modeled using the unstructured (UN) covariance structure. The least squares means (LSMs) estimated from the log-transformed observations were transformed back to the original scale using the exponential function. Median estimate on the original scale for each study lens was derived by this back transformation. For study lens comparison, the improvement percent (i.e., a percentage decrease relative to the control lens) was derived based on the model estimated medians and tested for statistical superiority with respect to each primary outcome.

## RESULTS

### Halo Size

**FIG. 5.**
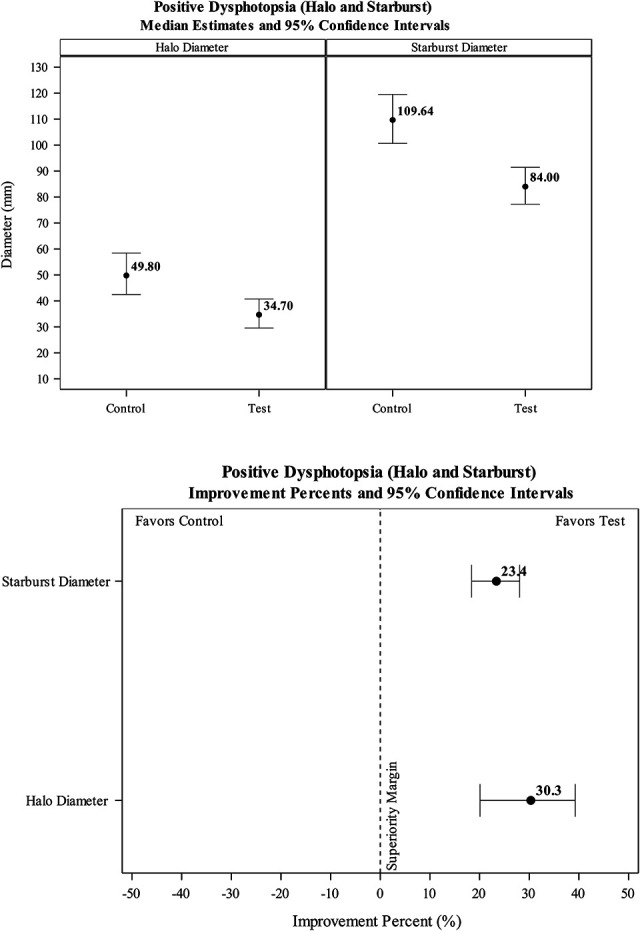
Halo and starburst diameter median estimates (upper panel) and percent improvements (lower panel).

Halo diameters were significantly smaller (*P*<0.001) in test eyes fitted with the HEV light absorbing test lens (M=39.73±15.72 mm) compared with test eyes fitted with the clear control lens (M=55.14±19.34 mm) (Table 1 and Fig. 5). As shown in Figure 5, the HEV-filtering lens significantly reduced the diameter of the halos by 30.3% (95% CI=20.1, 39.2).

**TABLE 1.** Descriptive Statistics and Results of Statistical Tests

	Test (N=61)	Control (N=61)
Halo (mm)		
Mean (SD)	39.73 (15.720)	55.14 (19.343)
Median	39.83	55.17
Model estimated median	34.70, 95% CI (29.54, 40.76)	49.80, 95% CI (42.44, 58.44)
Model estimated improvement percent (%)	30.3, 95% CI (20.1, 39.2), *P* <0.0001	
Starburst (mm)		
Mean (SD)	79.52 (20.079)	106.29 (30.871)
Median	76.67	102.00
Model estimated median	84.00, 95% CI (77.20, 91.40)	109.64, 95% CI (100.61, 119.49)
Model estimated improvement percent (%)	23.4, 95% CI (18.4, 28.1), *P* <0.0001	
Two-point threshold (403 nm)		
Mean (SD)	3.74 (1.843)	5.27 (1.923)
Median	3.08	4.86
Model estimated median	3.29, 95% CI (2.85, 3.79)	4.70, 95% CI (4.07, 5.44)
Model estimated improvement percent (%)	30.1, 95% CI (22.6, 36.9), *P* <0.0001	
Two-point threshold (broadband white)		
Mean (SD)	8.24 (4.593)	9.66 (4.877)
Median	7.39	8.72
Model estimated median	6.41, 95% CI (5.36, 7.68)	7.85, 95% CI (6.55, 9.42)
Model estimated improvement percent (%)	18.3, 95% CI (12.8, 23.6), *P* <0.0001	

N, total number of subjects; CI, confidence interval.

### Starburst Size

Starburst diameters were significantly smaller (*P*<0.001) in test eyes fitted with the HEV light absorbing filter (M=79.52±20.08 mm) compared with test eyes fitted with the clear control lens (M=106.29±30.87 mm) (Table 1 and Fig. 5). As shown in Figure 5, the HEV-filtering lens significantly reduced the diameter of the starbursts by 23.4% (95% CI=18.4, 28.1).

### Two-Point Thresholds

**FIG. 6.**
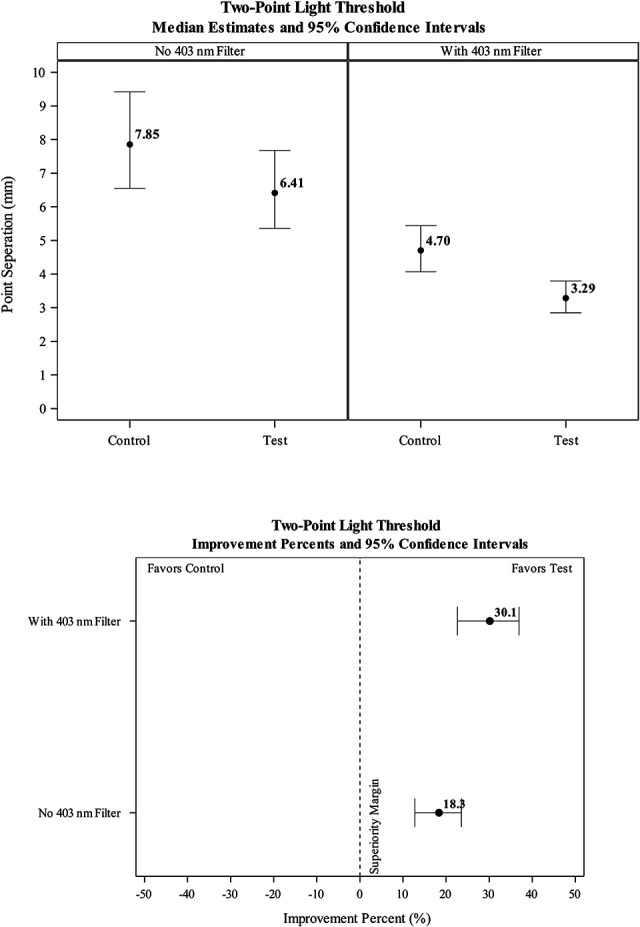
Two-point light threshold median estimates (upper panel) and percent improvements (lower panel).

Test eyes fitted with the HEV-filtering contact lens experienced significantly lower (*P*<0.0001), two-point thresholds in the broadband condition (8.24±4.59 mm) and the 403 nm condition (M=3.74±1.84 mm) than the clear control lens (9.66±4.88 mm, 5.27±1.92 mm in the broadband and 403 nm conditions, respectively) (Table 1 and Fig. 6). In the violet condition, threshold size between the HEV light filtering contact lens was 30.1% smaller than for the clear control (95% CI=22.6, 36.9). In the broadband condition, the HEV light filtering contact lens two-point threshold was 18.3% smaller than for the clear control (95% CI=12.8, 23.6; see Fig. 6).

## DISCUSSION

In this study, we compared the visual effects of a HEV-filtering contact lens that filters approximately 60% of HEV light (380–450 nm) to a control lens in the opposite eye that was relatively clear (absorbing approximately 10% of said light). All the testing was done using broadband xenon light chosen to emulate sunlight (except for an additional violet condition for two-point thresholds). We tested the diameter of halos and spokes generated by a small point of light several feet from the eye of the observer. We also measured the minimum distance necessary to perceive two small pinpoints of light as clearly distinct (i.e., the spread of light not overlapping). Across these measuring conditions, the HEV-filtering contact lenses in one eye performed better than the clear contact lens tested in the fellow eye. The magnitude of the differences ranged from approximately 18% to 30% improvements. The largest difference (30.1%) was for the stimulus that was the most strongly filtered, the violet two-point thresholds. As shown in Figure 2, light at 403 nm was strongly absorbed (>90% at the peak) by the HEV-filtering contact (compared with ∼5% for the clear).

McAliden et al.^27^ originally developed the Quality of Vision questionnaire to assess aspects of everyday vision that individuals found the most “bothersome.” This scale identified 10 major categories: glare, halos, starbursts, hazy vision, blurred vision, distortion, double vision, fluctuation, focusing difficulties, and depth perception. In this study, we found that a HEV-filtering contact lens can reduce halos and spokes by more than 20%. The refractive correction of the HEV-filtering and clear lenses were matched but our results also demonstrated a significant reduction in two-point thresholds (analogous to the Airy discs used as an indicator of resolution acuity). This finding suggested that the HEV-filtering lens also addresses some other categories that are bothersome such as hazy vision.

Because the test lens only filtered the shortest visible wavelengths, it also follows that this waveband may be driving much of the ocular aberrations that result in the perception of halos and spokes. Defocused HEV light, surprisingly, has minimal effects on many aspects of visual perception.^28^ Benedi-Garcia et al.^28^ concluded that the reason chromatic aberrations has minimal impact on visual function (despite being relatively extreme, almost 2 diopters, longitudinally, at 400 nm) is likely based on the system's ability to compensate. This compensation is accomplished by neural adaptation, limiting S-cone channel input,^29^ and through strategic filtering of short-wave light (e.g., by natural macular pigments^15,30^).

Despite all these corrective mechanisms, there are still many aspects of the visual stimulus subject to degradation, especially at the shortest visible wavelengths. For example, lower-order ocular aberrations (e.g., myopia, corneal astigmatism) still require external corrections. The results of this study suggest that the action spectrum for halos and spokes is similar to photophobia,^15^ exaggerated for short-wave visible light. The results of this study further suggest that filtering at short wavelengths can confer a visual advantage.
